# Research on Student’s T-Distribution Point Cloud Registration Algorithm Based on Local Features

**DOI:** 10.3390/s24154972

**Published:** 2024-07-31

**Authors:** Houpeng Sun, Yingchun Li, Huichao Guo, Chenglong Luan, Laixian Zhang, Haijing Zheng, Youchen Fan

**Affiliations:** 1 Graduate School, Space Engineering University, Beijing 101416, China; sunhoupeng@hgd.edu.cn (H.S.); lclzdfcs@hgd.edu.cn (C.L.); 2 Space Engineering University, Beijing 101416, China; zhanglaixian@126.com (L.Z.); 3120120251@bit.edu.cn (H.Z.); love193777@sina.com (Y.F.)

**Keywords:** point cloud registration, Student’s t-distribution mixture model, local features, adaptive penalties, composite weight coefficient

## Abstract

LiDAR offers a wide range of uses in autonomous driving, remote sensing, urban planning, and other areas. The laser 3D point cloud acquired by LiDAR typically encounters issues during registration, including laser speckle noise, Gaussian noise, data loss, and data disorder. This work suggests a novel Student’s t-distribution point cloud registration algorithm based on the local features of point clouds to address these issues. The approach uses Student’s t-distribution mixture model (SMM) to generate the probability distribution of point cloud registration, which can accurately describe the data distribution, in order to tackle the problem of the missing laser 3D point cloud data and data disorder. Owing to the disparity in the point cloud registration task, a full-rank covariance matrix is built based on the local features of the point cloud during the objective function design process. The combined penalty of point-to-point and point-to-plane distance is then added to the objective function adaptively. Simultaneously, by analyzing the imaging characteristics of LiDAR, according to the influence of the laser waveform and detector on the LiDAR imaging, the composite weight coefficient is added to improve the pertinence of the algorithm. Based on the public dataset and the laser 3D point cloud dataset acquired in the laboratory, the experimental findings demonstrate that the proposed algorithm has high practicability and dependability and outperforms the five comparison algorithms in terms of accuracy and robustness.

## 1. Introduction

Point cloud registration can be extensively applied in domains like 3D reconstruction [1,2,3], robotics [4,5,6,7], and computer vision [8,9,10,11,12,13,14]. Through rotation and translation transformation, point cloud registration joins three-dimensional point clouds from various viewpoints into a comprehensive data model. Iterative Closest Point (ICP) is the most classical algorithm for solving the point cloud registration problem, which takes the Euclidean distance between points and points as the objective function and uses iterative methods to optimize the registration. Nevertheless, finding the closest pair of points in the point cloud for each iteration of the ICP algorithm takes time, particularly in high-dimensional spaces or with enormous amounts of data. As a result, the ICP algorithm model has a lengthy iteration time, a high computing cost, and is prone to local optimum convergence. Researchers have refined the ICP algorithm structure in certain ways to enhance registration performance. By introducing overlap ratios, the Trimmed Iterative Closest Point (TrICP) algorithm [15] overcomes the registration problem of non-overlapping point sets. Scholars employ optimization algorithms and ICP algorithm to integrate and enhance the algorithm’s global performance to tackle the local convergence problem of point cloud registration [10,16,17]. To enhance the algorithm’s efficiency, the point features [10,18] and the local plane features [19,20] of the point cloud are introduced into the objective function of the registration, which provides an excellent initial attitude for the registration.

To increase the point cloud registration algorithm’s global robustness, statistical theory was incorporated into the registration process. A Coherent Point Drift (CPD) point cloud registration algorithm [21] was introduced by Andriy Myronenko. It implements the stiff registration and affine translation of the point cloud and formulates the registration problem as a probability density estimation function. To tackle the pairwise registration problem, researchers suggest the FilterReg algorithm [22] and GMMReg algorithm [23], which are based on the CPD algorithm as a framework. The FilterReg algorithm represents the three-dimensional set of points as a Gaussian mixture model (GMM) and then transforms the pairwise registration problem into a maximum likelihood estimation problem. GMMReg algorithm utilizes two GMMs to represent two point sets and describes the pairwise registration as a problem of aligning two GMMs, in which the expectation maximization (EM) algorithm minimizes the statistical difference measure between the two GMMs. Dylan proposed an SVR algorithm [24] that trains a one-class support vector machine with a Gaussian radial basis function kernel and subsequently approximates the output function with a Gaussian mixture model. Eckart proposed a tree-based Gaussian mixture model (GMMTree) algorithm [25]. This algorithm enhances the robustness of the algorithm by performing data association in logarithmic time and dynamically adjusting the level of detail to best match the complexity and spatial distribution characteristics of local scene geometry.

Pairwise registration can be accomplished with good accuracy and robustness using point cloud registration techniques based on GMM classes. Nevertheless, they are time-consuming due to the large number of point correspondences that need to be established. Additionally, these methods have a significant registration error when heavy-tail noise is present in the point cloud. Therefore, scholars propose a probability distribution using the Student’s t-distribution mixture model to construct point cloud registration [26,27,28,29]. This approach leverages Student’s t-distribution to produce registration results that are more reliable and accurate than GMM in situations where noise is present.

The 3D point clouds produced by LiDAR contain a variety of noises and outliers, and the laser point cloud data can be modelled using SMM even in the absence of prior noise distribution and missing outlier information. To increase point cloud registration technique’s resilience, this research suggests a Student’s t-distribution point cloud registration algorithm based on local features. The algorithm uses the normal information of the point cloud to calculate the local surface flatness, constructs the covariance matrix of the SMM according to the local features of the point cloud, and adaptively adds the registration penalty. When the local surface of the point cloud is relatively flat and the curvature changes little, a larger point-to-plane penalty should be added; on the contrary, when the local surface of the point cloud changes significantly, then larger point-to-point penalty should be added. Given the LiDAR imaging properties, the laser waveform is roughly Gaussian and the precision of the imaging edge area decreases as illumination brightness decreases. Consequently, we designed the weight coefficients of the SMM model and filtered out some outliers. Lastly, the objective function of the registration is solved using the EM algorithm.

The contents of this study are organized as follows. Related work is discussed in Section 2. SMM is used in Section 3 to define an objective function and point cloud registration model. In Section 4, the suggested approach is evaluated using six reference datasets as well as a lab-acquired laser 3D point cloud dataset. The outcomes are contrasted with those of five traditional algorithms. Section 5 provides a conclusion at the end.

## 2. Related Work

There are numerous point cloud registration techniques, and various techniques have been successfully used in numerous domains. In this study, research is conducted using a robust mixture model approach based on the properties of 3D point clouds. It is restricted to probabilistic methods for different types of mixture models in this review. A common method based on the GMM converts point cloud data and its features into a GMM, uses the parametric probability model of GMM to model the relationship between two point clouds. Then, uses parameter estimation and optimization algorithms to transform the model parameters into the best registration transformation [22,30,31,32,33,34,35]. Nevertheless, the GMM registration robustness of the laser 3D point cloud with complicated noise and outliers is low, and the point cloud registration approach based on the mixture model depends on the model having an excellent fitting effect on the point cloud. Ma Yanlin proposed a multi-viewpoint cloud registration based on SMM [28], which described all point clouds as an SMM distribution, and all SMM centroids were searched by neural network algorithms, improving efficiency. Min Zhe presented a method to model point direction using the von Mises–Fisher mixture model and realized the robust generalized point cloud registration [36]. For the 3D scanning laser point cloud registration problem, Shu Qin proposed a point cloud registration method based on the Laplace mixture model, which successfully overcame the nonlinear problem by using the sampling variance instead of the variance of the likelihood estimation [37]. To solve the issues of data loss and disorder during point cloud registration, Tang extended the point cloud mathematical model to the orthogonal factor model and used SMM to calculate the point cloud data to achieve fast real-time point cloud registration [38]. Alistair Barrie Forbes used a coordinate mixture model to fit the point cloud on the surface of the workpiece [39]. He constructed a feature correlation matrix based on the characteristics of the model, which improved the accuracy of the workpiece registration. In this paper, according to the characteristics of laser 3D point clouds, the SMM model will be used to carry out the research. The point cloud registration is constructed as a maximum likelihood probability function based on SMM, and the optimal registration of the point cloud is realized through optimal solving.

## 3. Method

### 3.1. Student’s T-Distribution Model

Sample data: $x=[x_{1},x_{2},\ldots ,x_{n}]$, assuming each data point obeys a n-dimensional Gaussian distribution, the probability density function of data x is defined as:

(1) $$f_{N}(x;\mu ,\Sigma )=\frac{1}{\sqrt{{\left(2\pi \right)}^{n}|\Sigma |}}e^{-\frac{1}{2}{\left(x-\mu \right)}^{T}\Sigma^{-1}(x-\mu )}$$

where μ is the mean of the Gaussian distribution and Σ is the covariance matrix of the Gaussian distribution.

The Gaussian distribution is susceptible to noises and outliers because most of the energy of the Gaussian probability density function is concentrated in the central area. Student’s t-distribution is more resilient and can exclude the interference of particular noises and outliers than the Gaussian distribution [40]. The following is an expression for the probability density function of Student’s t-distribution:

(2) $$f_{T}(x;\mu ,\Sigma ,v)=\frac{\Gamma \left(\frac{\nu +d}{2}\right)}{|\Sigma {|}^{\frac{1}{2}}\Gamma \left(\frac{\nu }{2}\right){\left(\pi \nu \right)}^{\frac{d}{2}}{\left[1+\frac{{\left(x-\mu \right)}^{T}\Sigma^{-1}(x-\mu )}{\nu }\right]}^{\frac{u+d}{2}}},$$

where $\Gamma \left(\cdot \right)$ denotes the gamma distribution and $\Gamma \left(\cdot \right)$ can be used as a priori information for Student’s t-distribution. Parameter v represents the tail degrees of freedom of Student’s t-distribution. 

Since Student’s t-distribution has a heavier tail than the Gaussian distribution, it can describe the point cloud more accurately by taking into account outliers and noises information. Changing the parameter v, the probability model of Student’s t-distribution will change the tail information weight, and the larger the v, the smaller the proportion of tail information. When v→∞, Student’s t-distribution is close to the Gaussian distribution.

### 3.2. SMM Point Cloud Registration Model

Point cloud registration is the process of using rotation and translation to register both the source and target point clouds. In general, the point cloud registration problem can be transformed into a mathematical problem to solve the maximum likelihood function. The transformed source point cloud is viewed as the SMM observation of the target point cloud by executing a rigid transformation on the source point cloud. The data in this article are described using the notation that follows: 

N—number of points in the source point cloud;

M—the number of points in the target point cloud;

$X\in R_{N\times 3}$—the source point cloud, the position of the *n*-th point in the source point cloud is $x_{n}$;

$Y\in R_{M\times 3}$—target point cloud, the position of the *m*-th point in the target point cloud is $y_{m}$;

$N\in R_{M\times 3}$—surface standard matrix, the *m*-th unit average vector in the point cloud Y is $n_{m}$;

I—Matrix of identity.

It is frequently necessary to include outlier distribution functions in GMM probabilistic models for point cloud registration. It is not necessary to include uniformly distributed outliers in the Student’s t-distribution model because it can handle noises and outliers quite effectively. The following is an expression of a probabilistic model based on Student’s t-distribution:

(3) $$p(x_{n})=\sum_{m=1}^{M}\pi (m)p(x_{n}|m)$$

where $x_{n}$ is the n-th point in the $X\in R_{N\times 3}$. The mathematical significance of $p(x_{n}|m)$ is the conditional probability that $x_{n}$ belongs to the component of the m-th Gaussian component and π(m) is the prior probability of $x_{n}$ being assigned to the m-th Gaussian component.

The likelihood that a point belongs to the M Gaussian components is the same in some mixture model methods [21,36], that is, π(m) = 1/M. When the detector’s imaging error is consistent within its detection range, this configuration makes sense. Nevertheless, noise, which is connected to the target’s imaging depth, has an impact on the camera’s measurement error [41,42]. Furthermore, the laser waveform of LiDAR imaging is Gaussian-like, the intermediate energy is higher than the edge energy, and the point cloud recovery accuracy in the center of the field of view is higher than that of the edge during 3D restoration. Thus, when creating the weight π(m), the algorithm should focus more on the alignment of the measured points with high confidence to enhance the mixture model’s capacity to represent the data. In this paper, based on the three-dimensional information of the point cloud, two main aspects are considered: the measurement error of the detector and the influence of the laser waveform on the recovery algorithm. The measurement error of the detector is related to the depth of the target. We set $\phi_{1}(x)={\mathrm{deep}}_{\min}/\mathrm{deep}(x)$, deep(x) as the error models are related to the distance of the target, ${\mathrm{deep}}_{\min}$ is the minimum measurement error within the detector range, and the error analysis and modelling of different detectors can be found in [42,43,44]. The recovery error of the laser waveform is mainly related to the distance between the point in the point cloud and the center of mass of the point cloud; the closer the distance, the higher the accuracy and the greater the weight, which can be expressed as $\phi_{2}(x)=\frac{1}{\sigma_{\mathrm{laser}}\sqrt{2\pi }}\exp(-\frac{{\left(x-x_{0}\right)}^{2}}{2\sigma_{\mathrm{laser}}{}^{2}})$, where $\sigma_{\mathrm{laser}}$ is the variance of the laser waveform and $x_{0}$ is the center of mass of the point cloud. The weight coefficient is expressed as the product of the two parts of the weight: $\phi (x)=\phi_{1}(x)\cdot \phi_{2}(x)$. Therefore, the weight factor of the mixture model is set to:

(4) $$\pi (m)=\frac{\phi (y_{m})}{\sum_{M}\phi (y_{m})}$$

The local feature information of the target surface is taken into account while developing the probability density function in order to increase the robustness and accuracy of point cloud registration. The CPD algorithm measures the registration effect by using the point-to-point Euclidean distance as the objective function. The point cloud registration solution is changed into an unsuitable optimization problem by the FilterReg algorithm, which uses the distance between the point and the plane as the objective function. This results in a non-full-rank problem in the probability density function. Thus, in this article, the combined penalty of point-to-point and point-to-plane distance is added as the objective function adaptively, and a full-rank covariance matrix is generated based on the local surface geometric properties of the point cloud. The following is an expression for the intended probability density function:

(5) $$\begin{array}{c} p(x_{n}|m)=c_{m}\frac{\Gamma \left(\frac{\nu +d}{2}\right)}{|\Sigma_{m}{|}^{\frac{1}{2}}\Gamma \left(\frac{\nu }{2}\right){\left(\pi \nu \right)}^{\frac{d}{2}}{\left[1+\frac{{\left(x_{n}-y_{m}\right)}^{T}\Sigma_{m}^{-1}(x_{n}-y_{m})}{\nu }\right]}^{\frac{u+d}{2}}}\\ \Sigma_{m}^{-1}=\frac{1}{\sigma^{2}}(\alpha_{m}n_{m}n_{m}^{T}+I) \end{array}$$

$c_{m}$ is a normalized constant and $\sigma^{2}$ is a covariance multiplier that can be expressed as:

(6) $$\sigma^{2}=\frac{\sum_{n=1}^{N}\sum_{m=1}^{M}P_{mn}∥g(x_{n})-y_{m}{∥}_{{\left(\alpha_{m}n_{m}n_{m}^{T}+I\right)}^{-1}}^{2}}{3\sum_{n=1}^{N}\sum_{m=1}^{M}P_{mn}}$$

$\Sigma_{m}$ is the covariance matrix constructed in this paper, and its inverse matrix $\Sigma_{m}^{-1}$ is composed of a linear combination of the identity matrix I and $n_{m}n_{m}^{T}$. In Equation (6), the physical meaning ${\left(x_{n}-y_{m}\right)}^{T}I(x_{n}-y_{m})$ is the distance between point $x_{n}$ in point cloud X and point $y_{m}$ in point cloud Y, and the Euclidean distance from point-to-point is taken as the objective function to measure the registration effect. The physical meaning ${\left(x_{n}-y_{m}\right)}^{T}\frac{1}{\sigma^{2}}\alpha_{m}n_{m}n_{m}^{T}(x_{n}-y_{m})$ is the distance between a point $x_{n}$ in point cloud X and the local plane around a point $y_{m}$ in point cloud Y. Therefore, this paper designs the covariance matrix $\Sigma_{m}^{-1}$ to describe the point-to-point distance between points $x_{n}$ and points $y_{m}$ and the distance from a point $x_{n}$ to the local plane around the point $y_{m}$.

In this paper, the point-to-plane penalty is added adaptively based on the local surface flatness of the point cloud. The surface variation parameter $\kappa_{m}$ describes the deviation of a point in the point cloud from its tangent and can be evaluated by evaluating the surface variation $\kappa_{m}$ near the point $y_{m}$, the design penalty factor, and the surface variation $\kappa_{m}\in (0,1/3)$. According to the properties of the parameter $\kappa_{m}$, a function $\alpha_{m}$ is designed as a penalty coefficient in the probability density function:

(7) $$\alpha_{m}=\frac{1-3*\kappa_{m}*\exp\left({\left(3-\frac{1}{\kappa_{m}}\right)}^{\lambda }\right)}{1+3*\kappa_{m}*\exp\left({\left(3-\frac{1}{\kappa_{m}}\right)}^{\lambda }\right)}\alpha_{\max}$$

where $\alpha_{\max}$ is the upper bound of the penalty coefficient and λ is the coefficient that controls the sensitivity of $\alpha_{m}$ to $\kappa_{m}$. When $\kappa_{m}$ → 1/3 and $\alpha_{m}$ → 0, the surface of the point cloud changes incredibly. When $\kappa_{m}$ → 0 and $\alpha_{m}$ → $\alpha_{\max}$, the surface of the point cloud changes little and tends to be flat. The adaptive modulation coefficient $\alpha_{m}$ is set according to the point cloud’s surface characteristics. When the surface of the point cloud is flat, a sizeable point-to-plane penalty coefficient is set. When the surface curvature is large or the noise is damaged, the point-to-plane penalty and the matching error are reduced.

### 3.3. Solve the Objective Function

Suppose the point cloud registration’s transformation function is g(⋅), the point $x_{n}$ can be represented as $g(x_{n})$ after transformation. The likelihood function of the registration can be expressed as:

(8) $$\begin{array}{cc} L\left(s,\sigma^{2}\right) & =-\sum_{n=1}^{N}\log\left(p\left(g\left(x_{n}\right)\right)\right)\\ & =-\sum_{n=1}^{N}\log(\sum_{m=1}^{M}\pi (m)p((g(x_{n})|m)) \end{array}$$

This paper uses the EM algorithm to solve the maximum likelihood estimation problem. In the EM framework, the complete data include the observed variable $x_{n}$ and the latent variable $z_{mn}$, and the likelihood function for solving the problem can be expressed as:

(9) $$\begin{array}{cc} L_{c}(g,\sigma^{2},Z) & =-\log\prod_{n=1}^{N}(\prod_{m=1}^{M}{\left(\pi (m)p(g(x_{n})|m)\right)}^{z_{mn}})\\ & =-\sum_{n=1}^{N}(\sum_{m=1}^{M}z_{mn}\log(\pi (m)p(g(x_{n})|m))) \end{array}$$

Since the latent variable $z_{mn}$ cannot be directly observed, the posterior probability of the latent variable can be calculated according to the Bayesian principle:

(10) $$\begin{array}{cc} P(z_{mn}=1|g_{old}(x_{n})) & =\frac{P(z_{mn}=1)p(g_{old}(x_{n})|z_{mn=1})}{p(g_{old}(x_{n}))}\\ & =\frac{\pi (m)p(g_{old}(x_{n}|m))}{\sum_{M}\pi (m)p(g_{old}(x_{n})|m)} \end{array}$$

Therefore, the objective function of point cloud registration can be expressed as:

(11) $$\begin{array}{cc} Q & =-\sum_{n=1}^{N}\sum_{m=1}^{M}E(z_{mn}|g_{old}(x_{n}))\log\left(p(g(x_{n})|m)\right)\\ & =-\sum_{n=1}^{N}\sum_{m=1}^{M}[1\cdot P(z_{mn}=1|g_{old}(x_{n}))+0\cdot P(z_{mn}=0|g_{old}(x_{n}))]\log\left(p(g(x_{n})|m)\right)\\ & =-\sum_{n=1}^{N}\sum_{m=1}^{M}P_{mn}(\log(c_{m})-\frac{1}{2}∥g(x_{n})-y_{m}{∥}_{\Sigma_{m}^{-1}}^{2}) \end{array}$$

where $c_{m}$ is the normalization constant, $\Sigma_{m}$ is the covariance matrix, and $P_{mn}$ can be expressed as:

(12) $$P_{mn}=\frac{\pi \left(m\right)p\left(g_{\mathrm{old}\;}\left(x_{n}\right)|m\right)}{\sum_{M}\pi \left(m\right)p\left(g_{old}\left(x_{n}\right)|m\right)}$$

The best point cloud registration transformation matrix can be obtained by solving the target likelihood function of point cloud registration to realize the operation.

## 4. Experiment

We tested the suggested technique on both the laboratory’s laser 3D point cloud data and the public dataset to ensure its resilience and correctness. Every experiment was run on a personal computer (PC) equipped with an A6000 GPU and an Intel(R) Xeon(R) Silver 4210RCPU. The authors of the studies or open-source libraries provided comparison methods. In the experiment, the algorithm parameters were provided by the author or the program; if not, we carefully adjusted them. To reduce the impact of unintentional errors, the experimental findings were obtained by averaging them after repeated experiments and deleting the outliers.

### 4.1. Point Cloud Registration of Public Datasets

In this section, we introduce noises and outliers to the dataset to evaluate the registration algorithm’s performance. Here, the term “outlier” refers to a specific percent of Gaussian random noise applied to the point cloud’s exterior space, and the term “noise” refers to a specific percent of Gaussian randomization of the point cloud’s points. The suggested approach was used to register the public dataset after introducing noises and outliers to the point cloud. The algorithm was then tested and evaluated using the registration results.

We set the maximum size of the point cloud in the x, y, and z dimensions to 2 mm and sample all the point clouds to 3000 points to guarantee the similarity of the density of the point cloud dataset. We configured the point cloud’s rotation angle to be randomly generated in three directions, but we made sure that the point cloud’s rotation angles in the x, y, and z axes added up to 60°. Likewise, we designated the point cloud’s translation distance in three directions to be created at random. However, we needed to make sure that the sum of the translation distance of the point cloud in the x, y, and z directions is 6 mm. Using the identical parameter values, we compare the registration method described in this paper with the following algorithms: FilterReg algorithm, Support Vector Registration (SVR) algorithm, GMMTree algorithm, TrICP algorithm, and CPD algorithm. After the source point cloud is input in 3D space, the target point cloud is obtained after the rotation and translation transformation of the same angle, and then the source point cloud and the target point cloud are registered by the algorithm. Thirty independent experiments were carried out for each group of point clouds, and the experimental results were averaged as the results for error evaluation. We use the average distance error $D_{\mathrm{error}}$ of the points in the point concentration and the angular error $A_{\mathrm{error}}$ of the rotation matrix to measure the point cloud registration effect. The two error evaluation functions can be expressed as:

(13) $$\left\{\begin{array}{l} D_{\mathrm{error}}=\frac{1}{N}\sum_{i=1}^{N}{\left\|g_{est}(x_{i})-g_{gt}(x_{i})\right\|}_{{}_{2}}\\ A_{\mathrm{error}}=a\cos((trace(R_{gt}{}^{-1}R_{est})-1)/2) \end{array}\right.$$

where $x_{i}$ is the *i*-th point in the source point set and $g_{est}(x)$ and $g_{gt}(x)$ are the estimated transformation matrix and the real transformation matrix of point cloud registration, respectively. $R_{gt}$ and $R_{est}$ are the actual and estimated rotation matrices for point cloud registration, respectively.

**Figure 1:**
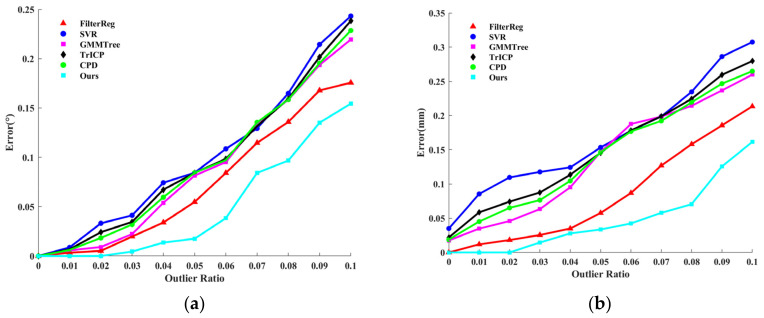
Point cloud registration error with different Gaussian outlier ratios. (**a**) the point average distance error of the algorithmic registration. (**b**) the angular error of the rotation matrix for the registration algorithm.

**Figure 2:**
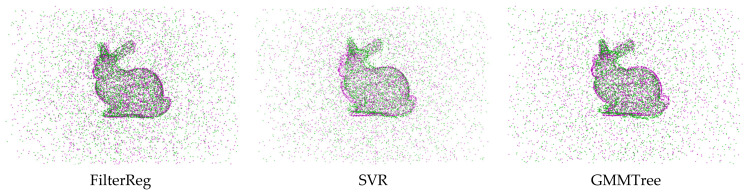

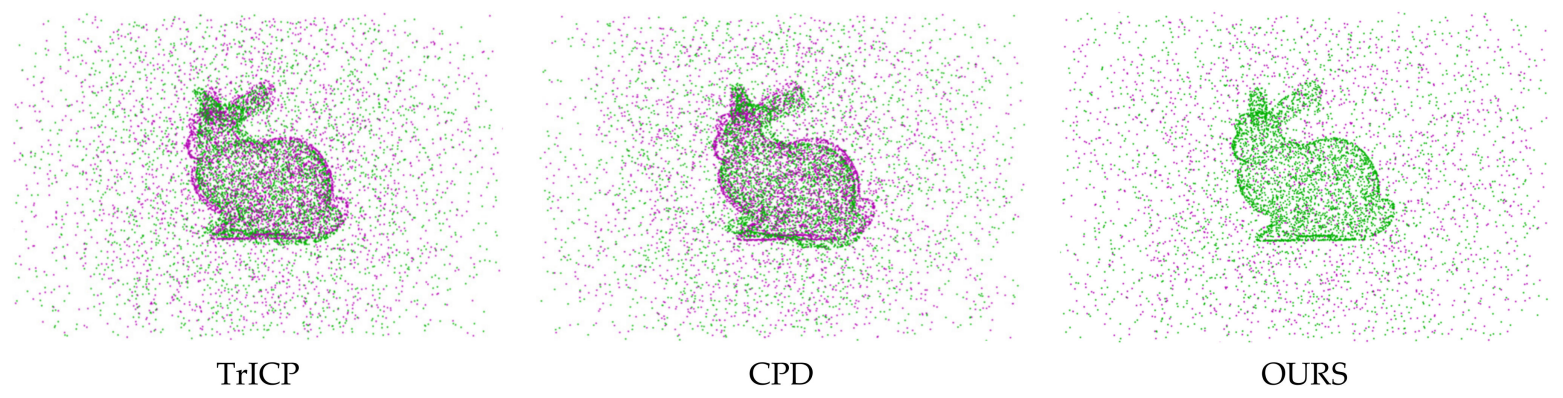
The point cloud registration results when the Gaussian outlier ratio = 1.

In the outlier experiment, we modified the source and target point cloud data by adding varying amounts of Gaussian outlier noises. When Gaussian outliers are included, Figure 1 displays the point cloud registration error results; Figure 1a shows the point average distance error $D_{\mathrm{error}}$ of the algorithmic registration. Figure 1b illustrates the angular error $A_{\mathrm{error}}$ of the rotation matrix for the registration algorithm. In Figure 1, as the proportion of outliers increases, the distance and angle errors of the registration increase. The error curve shows that the registration error of the algorithm proposed in this paper is the smallest, and the registration accuracy is the highest. Figure 2 shows the registration results of the registration algorithm at the Gaussian outlier ratio = 1. From a subjective visual point of view, the registration results of the SVR algorithm, GMMTree algorithm, TrICP algorithm, and CPD algorithm are close to each other and the error rate is high. The algorithm we proposed in this paper has the best registration effect.

**Figure 3:**
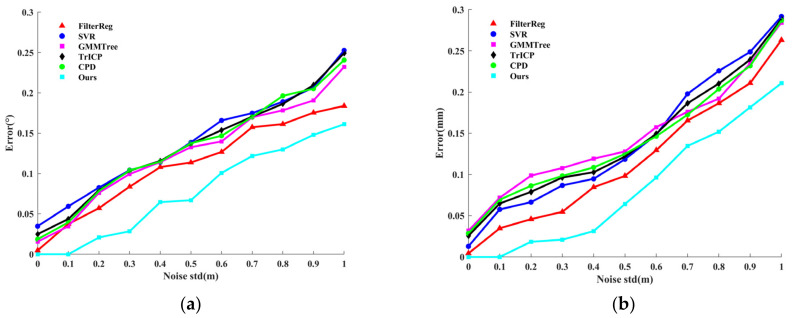
Point cloud registration error with different Gaussian noise ratios. (**a**) the point average distance error of the algorithmic registration. (**b**) the angular error of the rotation matrix for the registration algorithm.

**Figure 4:**
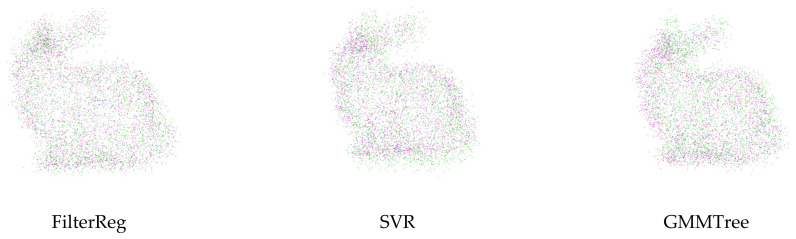

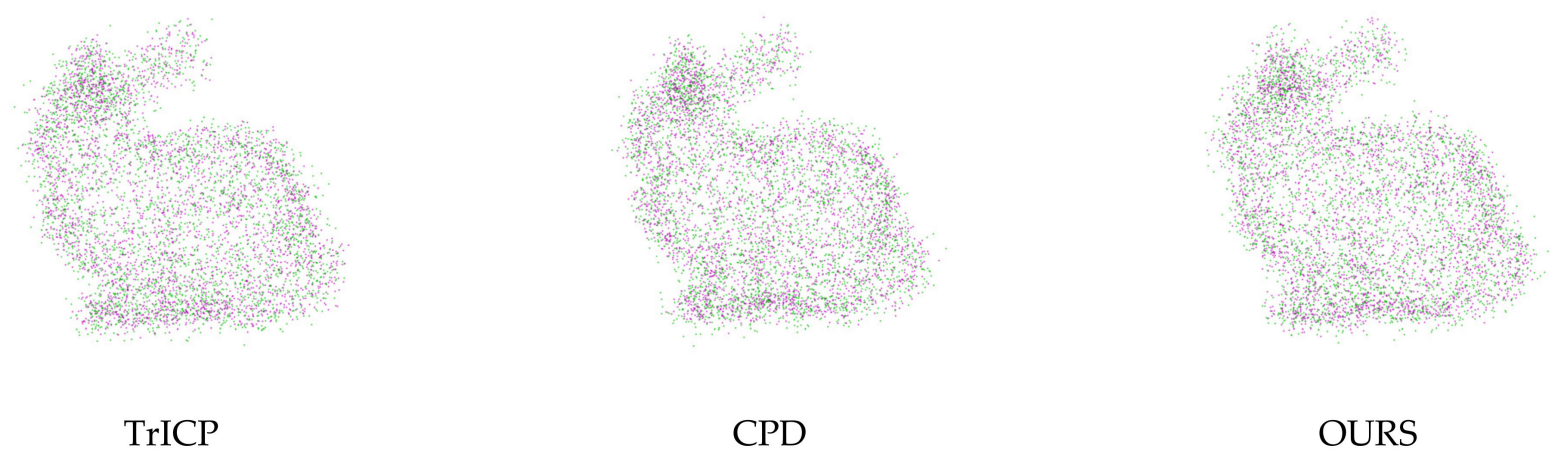
The point cloud registration result when the Gaussian noise ratio is 0.04.

In the noise experiment, we destroy the source and target point clouds by applying varying ratios of Gaussian noises. The results of point cloud registration with Gaussian noise applied are displayed in Figure 3. Figure 3a shows the point mean distance error $D_{\mathrm{error}}$ for the algorithm registration; Figure 3b illustrates the angular error $A_{\mathrm{error}}$ of the rotation matrix for the registration algorithm. As can be seen in the experimental results, with the increase of Gaussian noise, the performance of the algorithm we proposed in this paper is significantly better than that of the other five algorithms, and it has good robust performance. Figure 4 shows the registration results of the registration algorithm when the Gaussian noise ratio = 0.04, and subjectively, the algorithm we proposed in this paper can overcome the influence of noise and achieve accurate registration.

Computation complexity is another crucial metric for assessing the algorithm’s performance, in addition to registration correctness. There are two types of computation complexity for algorithms: time complexity and spatial complexity. The relationship between the amount of input data and the time it takes to execute an algorithm is known as time complexity. We vary the number of points input to the point cloud and measure the time required by the main method for point cloud registration to verify the time complexity of the algorithm. After testing, when the amount of input point cloud data is doubled, the algorithm time increases by four times, so we determine the algorithm’s time complexity is $O(n^{2})$. The amount of memory space used by an algorithm while it is in operation is measured by its spatial complexity, which in this work is related to three aspects: the storing of input data, the storage of local features, and the computation of the objective function. The two point cloud datasets are the algorithm’s input, the storage space of each point cloud is O(N⋅d), *N* is the number of points of the point cloud, and *d* is the dimension of points. In the calculation process, the algorithm needs to use the feature data of the point cloud, including curvature and average vector. This part of the storage space is O(N⋅f), and *f* is the dimension of the eigenvector. In the calculation process of the objective function, intermediate variables such as weight matrix and distance matrix need to be stored and are usually related to the size and feature dimension of the point cloud. In general, the spatial complexity of the algorithm can be approximated by O(N⋅d)+O(N⋅f). Therefore, the spatial complexity of the algorithm is on the order of O(n). Table 1 and Table 2 provide the time taken for point cloud registration of the dataset bunny at different outlier ratios and noise ratios, respectively.

The algorithm presented in this research has a high computing efficiency and a rapid operation speed based on its time complexity and running time.

Determining the boundary case of algorithm failure and providing success cases are essential for gauging an algorithm’s overall performance. We set the translation distances in the x, y, and z directions to a total of 6 mm and the sum of the point cloud’s rotation angles in the x, y, and z directions to 60°. The point cloud registration performs well with this option set.

**Figure 5:**
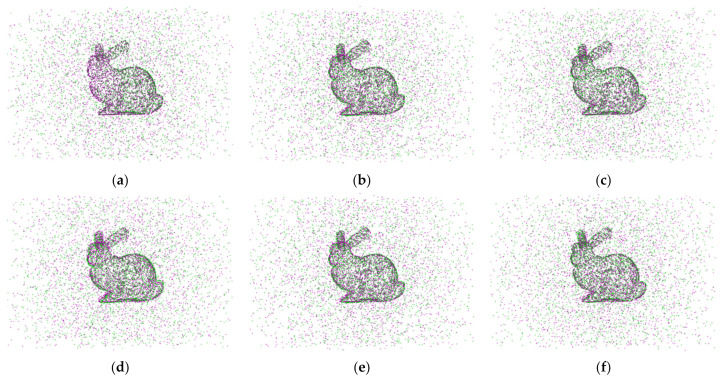
When outlier noise ratios = 1, the point cloud registration results in different sums of the translation distances. The sum of the translation distances for each subgraph is: (**a**) = 10 mm; (**b**) = 15 mm; (**c**) = 20 mm; (**d**) = 25 mm; (**e**) = 30 mm; (**f**) = 35 mm.

We set the sum of the point cloud’s rotation angles in the x, y, and z axes to 60° to quantify the impact of the translation distance on the point cloud’s registration. The point cloud’s translation distance in the x, y, and z directions adds up to 10 mm–35 mm. The point cloud registration results are displayed in Figure 5 with the point cloud translation distance spacing of Figure 5a–f being 5 mm apart in the case of outlier noise = 1. Table 3 displays the point cloud registration error.

**Figure 6:**
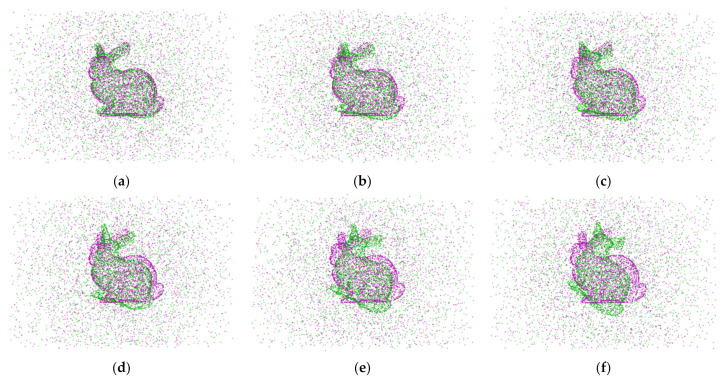
When outlier noise ratios = 1, the point cloud registration results at different sums of the rotation angle. The sum of the rotation angle for each subgraph is: (**a**) = 70°; (**b**) = 80°; (**c**) = 90°; (**d**) = 100°; (**e**) = 110°; (**f**) = 120°.

We set the rotation angle of the point cloud in the x, y, and z directions to be 70–120° and the total of the translation distances of the point cloud in the x, y, and z directions to 6 mm to quantify the impact of the rotation angle on the registration of the point cloud. Figure 6 displays the point cloud registration results when the outlier noise = 1, with a 10° separation between the point cloud rotation angle intervals of Figure 6a,f. Table 4 displays the point cloud registration error.

The registration results show that the translation distance of the point cloud has little effect on the registration accuracy of the suggested approach and that it can be disregarded. The point cloud’s rotation angle has a big impact on the algorithm’s accuracy. The target’s registration accuracy starts to vary dramatically when the total of the point cloud’s rotation angles in the x, y, and z directions exceeds 90°.

**Figure 7:**
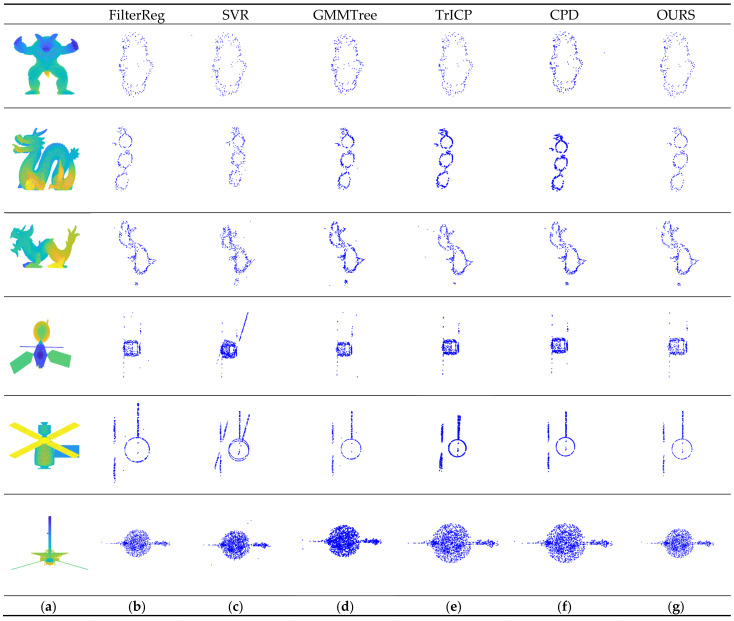
Point cloud registration cross-sectional view: (**a**) aligned 3D model; (**b**) the results of the FilterReg algorithm; (**c**) the results of the SVR algorithm; (**d**) the results of the GMMTree algorithm; (**e**) the results of the TrICP algorithm; (**f**) the results of the CPD algorithm; and (**g**) the results of our research.

To minimize the impact of datasets on algorithm registration outcomes, this research chooses many sets of datasets for registration. The registration data are selected from the publicly available Stanford University 3D scan repository [45], and spacecraft models are available on the National Aeronautics and Space Administration (NASA) website. Figure 7 shows the registration results according to the cross-section to facilitate subjective comparison and judgment of the registration effect. As can be seen from Figure 7, the proposed algorithm achieves the best registration effect on all datasets.

To evaluate the robustness of the suggested technique, we registered two datasets with Gaussian noise. Thirty independent tests were conducted for each group of experiments, and the datasets of the two sets of tests were supplemented with 40% and 50% of the Gaussian noise data points, respectively. Table 5 and Table 6 display the outcomes of the experiment.

The best registration outcomes for every set of experiments are bolded in Table 5 and Table 6. For point cloud registration tests utilizing various datasets, experimental results demonstrate that the technique suggested in this study has good robustness and registration accuracy when Gaussian noise is included.

### 4.2. Laser 3D Point Cloud Registration

**Figure 8:**
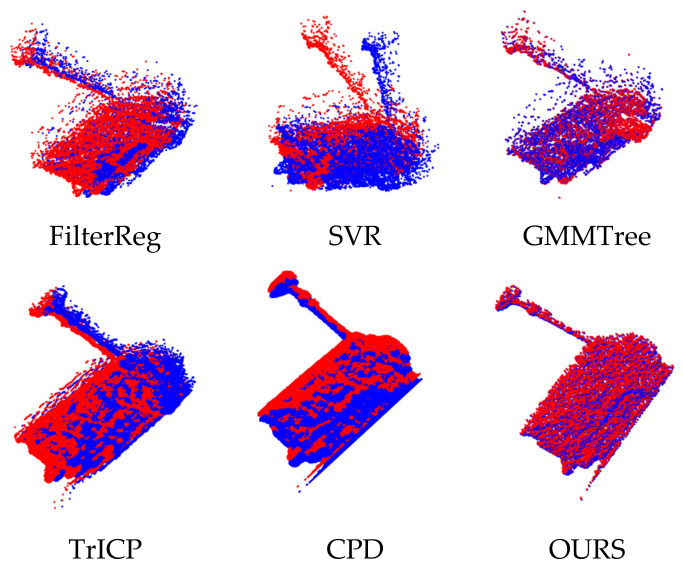
Laser 3D point cloud registration results.

In Section 4.1, we used the algorithm to register the public dataset, and our algorithm showed good competitiveness in both the original dataset and the dataset with added noise. Due to its imaging principle, laser 3D point clouds include speckles and Gaussian noise. This section uses the laser 3D point cloud data obtained by the laboratory LiDAR for registration after preprocessing operations such as target extraction and noise reduction. The experimental results are shown in Figure 8.

In Figure 8, the registration error of the SVR algorithm is significant, and the central part of the target can achieve partial registration. In contrast, the columnar region in the target has a significant deviation, and the registration accuracy is low. The FilterReg algorithm, GMMTree algorithm, TrICP algorithm, and CPD algorithm can realize the registration of most point clouds. However, due to the influence of noise, there is a specific registration error. From the perspective of visual subjectivity, the algorithm proposed in this paper can achieve the best point cloud registration.

Table 7 shows the registration errors for the six algorithms. The registration’s rotation and translation errors of the SVR algorithm are the largest, which is more suitable for the coarse registration work before accurate registration. The registration errors of the FilterReg algorithm, GMMTree algorithm, TrICP algorithm, and CPD algorithm are small, and the registration accuracy is disturbed by noise, which has certain limitations for the application scenarios of high-precision registration. The algorithm proposed in this paper has the highest registration accuracy and can realize the accurate registration task of laser 3D point cloud.

This section uses five registration algorithms to calculate the point cloud registration results under different datasets and noise environments. We are judging from the algorithm’s registration results. Compared with other related algorithms, such as the CPD algorithm, FilterReg algorithm, and GMMTree algorithm, the proposed algorithm has apparent advantages in registration accuracy and computation efficiency, which proves its effectiveness.

## 5. Conclusions

In this paper, a novel Student’s t-distribution point cloud registration algorithm based on local features is proposed. The algorithm builds an objective function using the Student’s t-distribution mixture model and solves the problem using the maximum likelihood function. Six publicly available datasets are tested to confirm the algorithm’s performance. This research presents a comparison of five different point cloud registration algorithms. The point cloud registration algorithms that are suggested have clear advantages in terms of efficiency and accuracy of registration. Lastly, we performed the registration test using the laser 3D point cloud that was gathered in the lab. According to experimental data, the suggested method performs well in terms of efficiency and accuracy. This algorithm’s successful application offers a workable solution to the laser 3D point cloud registration problem. Its high practicability and dependability can also strongly promote future research and applications in related fields.

## Figures and Tables

**Table 1:** Run time of point cloud registration at different outlier ratios.

Outlier ratios	0.1	0.2	0.3	0.4	0.5	0.6	0.7	0.8	0.9	1
Time (s)	0.7176	0.7518	0.8027	0.7951	0.8323	0.8546	0.8742	0.9074	0.9415	0.9289

**Table 2:** Run time of point cloud registration at different noise ratios.

Noise ratios	0.01	0.02	0.03	0.04	0.05	0.06	0.07	0.08	0.09	0.1
Time (s)	0.3959	0.4273	0.4574	0.4866	0.5387	0.5218	0.5749	0.5782	0.6075	0.6169

**Table 3:** When outlier noise ratios = 1, the registration error at different sums of the translation distances.

The Sum of the Point Cloud Translation Distance (mm)	10	15	20	25	30	35
A_error_	0.2123	0.2239	0.2451	0.2324	0.2547	0.2356
D_error_	0.1675	0.1735	0.1864	0.1758	0.1975	0.2147

**Table 4:** When outlier noise ratios = 1, the registration error at different sums of the rotation angle.

The Sum of the Point Cloud Rotation Angle (°)	70°	80°	90°	100°	110°	120°
A_error_	0.2955	0.3478	0.4971	0.5308	0.6295	0.8530
D_error_	0.2546	0.2764	0.2964	0.3547	0.5741	0.7828

**Table 5:** Point cloud registration error results with 40% Gaussian noise added.

	FilterReg		SVR		GMMTree		TrICP		CPD		OURS	
	D_error_	A_error_	D_error_	A_error_	D_error_	A_error_	D_error_	A_error_	D_error_	A_error_	D_error_	A_error_
Armadillo	0.0523	0.0085	0.0617	0.1152	0.0543	0.1034	0.0594	0.1107	0.0572	0.1087	**0.0387**	**0.0048**
Dragon	0.0627	**0.0065**	0.0707	0.0762	0.0634	0.0681	0.0684	0.0756	0.0673	0.0725	**0.0413**	0.0066
Long	0.0829	0.0225	0.0874	0.1135	0.0861	0.0947	0.0868	0.1023	0.0862	0.0987	**0.0514**	**0.0133**
MRO4	0.0648	0.0123	0.1084	0.0312	0.0543	0.0289	0.0842	0.0305	0.0769	0.0298	**0.0338**	**0.0081**
SKYLAB	0.0418	0.0087	0.0939	0.0230	0.0697	0.0364	0.0764	0.0261	0.0751	0.0346	**0.0329**	0.0025
Voyager	0.0991	0.0147	0.1074	0.0287	0.0967	0.0271	0.0985	0.0274	0.0982	0.0295	**0.0501**	**0.0075**

**Table 6:** Point cloud registration error results with 50% Gaussian noise added.

	FilterReg		SVR		GMMTree		TrICP		CPD		OURS	
	D_error_	A_error_	D_error_	A_error_	D_error_	A_error_	D_error_	A_error_	D_error_	A_error_	D_error_	A_error_
Armadillo	0.0685	0.0263	0.0857	0.1452	0.0741	0.0843	0.07658	0.0962	0.7692	0.0861	**0.0635**	**0.0067**
Dragon	0.0801	0.0148	0.0868	0.1097	**0.0695**	0.0942	0.07214	0.1006	0.7034	0.9657	0.0697	**0.0083**
Long	0.0964	0.0357	0.1041	0.1246	0.0894	0.1108	0.0965	0.1163	0.9231	0.1135	**0.0717**	**0.0173**
MRO4	0.0851	0.0201	0.1353	0.0638	0.0946	0.0473	0.1203	0.0528	0.1108	0.4854	**0.0738**	**0.0088**
SKYLAB	0.0643	0.0163	0.1142	0.0519	0.1023	0.0407	0.1068	0.0467	0.1052	0.4263	**0.0572**	**0.0082**
Voyager	0.1087	0.0919	0.1269	0.0491	0.1139	0.0645	0.1162	0.5362	0.1148	0.5739	**0.0879**	**0.0198**

**Table 7:** Evaluation of laser 3D point cloud registration.

	FilterReg	SVR	GMMTree	TrICP	CPD	OURS
D_error_	0.0136	0.0409	0.0287	0.0336	0.2947	**0.0084**
A_error_	0.0347	0.0369	0.0398	0.0384	0.03762	**0.0021**

## Data Availability

The data that support this study are proprietary and may only be provided with restrictions.

## References

[1] Dong Z., Yang B., Liang F., Huang R., Scherer S. (2018). Hierarchical registration of unordered TLS point clouds based on binary shape context descriptor. ISPRS J. Photogramm. Remote Sens..

[2] Choi S., Zhou Q.-Y., Koltun V. Robust reconstruction of indoor scenes. Proceedings of the IEEE Conference on Computer Vision and Pattern Recognition.

[3] Henry P., Krainin M., Herbst E., Ren X., Fox D. (2012). RGB-D mapping: Using Kinect-style depth cameras for dense 3D modeling of indoor environments. Int. J. Robot. Res..

[4] Yu F., Xiao J., Funkhouser T. Semantic alignment of LiDAR data at city scale. Proceedings of the IEEE Conference on Computer Vision and Pattern Recognition.

[5] Wen C., Tan J., Li F., Wu C., Lin Y., Wang Z., Wang C. (2021). Cooperative indoor 3D mapping and modeling using LiDAR data. Inf. Sci..

[6] Li A., Wang J., Xu M., Chen Z. (2021). DP-SLAM: A visual SLAM with moving probability towards dynamic environments. Inf. Sci..

[7] Hasanvand M., Nooshyar M., Moharamkhani E., Selyari A. (2023). Machine learning methodology for identifying vehicles using image processing. Artif. Intell. Appl..

[8] Yang J., Li H., Campbell D., Jia Y.J. (2015). Go-ICP: A globally optimal solution to 3D ICP point-set registration. IEEE Trans. Pattern Anal. Mach. Intell..

[9] Ma J., Zhao J., Jiang J., Zhou H., Guo X. (2019). Locality preserving matching. Int. J. Comput. Vis..

[10] Sandhu R., Dambreville S., Tannenbaum A. (2009). Point set registration via particle filtering and stochastic dynamics. IEEE Trans. Pattern Anal. Mach. Intell..

[11] Deng W., Cai X., Wu D., Song Y., Chen H., Ran X., Zhou X., Zhao H. (2024). MOQEA/D: Multi-objective QEA with decomposition mechanism and excellent global search and its application. IEEE Trans. Intell. Transp. Syst..

[12] Li M., Wang Y., Yang C., Lu Z., Chen J. (2024). Automatic diagnosis of depression based on facial expression information and deep convolutional neural network. IEEE Trans. Comput. Soc. Syst..

[13] Nsugbe E. (2023). A pilot on the use of unsupervised learning and probabilistic modelling towards cancer extent prediction. Artif. Intell. Appl..

[14] Sun Q., Chen J., Zhou L., Ding S., Han S. (2024). A study on ice resistance prediction based on deep learning data generation method. Ocean Eng..

[15] Chetverikov D., Stepanov D., Krsek P. (2005). Robust Euclidean alignment of 3D point sets: The trimmed iterative closest point algorithm. Image Vis. Comput..

[16] Tian Y., Yue X., Zhu J. (2023). Coarse–Fine Registration of Point Cloud Based on New Improved Whale Optimization Algorithm and Iterative Closest Point Algorithm. Symmetry.

[17] Saleh A.R., Momeni H.R. (2024). An improved iterative closest point algorithm based on the particle filter and K-means clustering for fine model matching. Vis. Comput..

[18] Lei H., Jiang G., Quan L. (2017). Fast descriptors and correspondence propagation for robust global point cloud registration. IEEE Trans. Image Process..

[19] Wang X., Li Y., Peng Y., Ying S. (2020). A coarse-to-fine generalized-ICP algorithm with trimmed strategy. IEEE Access.

[20] Serafin J., Grisetti G. NICP: Dense normal based point cloud registration. Proceedings of the 2015 IEEE/RSJ International Conference on Intelligent Robots and Systems (IROS).

[21] Myronenko A., Song X. (2010). Point set registration: Coherent point drift. IEEE Trans. Pattern Anal. Mach. Intell..

[22] Gao W., Tedrake R. Filterreg: Robust and efficient probabilistic point-set registration using gaussian filter and twist parameterization. Proceedings of the IEEE/CVF Conference on Computer Vision and Pattern Recognition.

[23] Jian B., Vemuri B.C. (2010). Robust point set registration using gaussian mixture models. IEEE Trans. Pattern Anal. Mach. Intell..

[24] Campbell D., Petersson L. An adaptive data representation for robust point-set registration and merging. Proceedings of the IEEE International Conference on Computer Vision.

[25] Eckart B., Kim K., Kautz J. (2018). Fast and accurate point cloud registration using trees of gaussian mixtures. arXiv.

[26] Hou Z., Tu J., Geng C., Hu J., Tong B., Ji J., Dai Y. (2018). Accurate and robust non-rigid point set registration using student’st mixture model with prior probability modeling. Sci. Rep..

[27] Yang L., Yang Y., Wang C., Li F. (2023). Rotation robust non-rigid point set registration with Bayesian student’st mixture model. Vis. Comput..

[28] Ma Y., Zhu J., Tian Z., Li Z. (2022). Effective multiview registration of point clouds based on Student’st mixture model. Inf. Sci..

[29] Ma Y., Zhu J., Li Z., Tian Z., Li Y. (2020). Effective multi-view registration of point sets based on student’s t mixture model. arXiv.

[30] Eckart B., Kim K., Kautz J. Hgmr: Hierarchical gaussian mixtures for adaptive 3d registration. Proceedings of the European Conference on Computer Vision (ECCV).

[31] Granger S., Pennec X. Multi-scale EM-ICP: A fast and robust approach for surface registration. Proceedings of the Computer Vision—ECCV 2002: 7th European Conference on Computer Vision.

[32] Min Z., Wang J., Meng M.Q.-H. (2019). Robust generalized point cloud registration with orientational data based on expectation maximization. IEEE Trans. Autom. Sci. Eng..

[33] Ravikumar N., Gooya A., Frangi A.F., Taylor Z.A. Generalised coherent point drift for group-wise registration of multi-dimensional point sets. Proceedings of the Medical Image Computing and Computer Assisted Intervention—MICCAI 2017: 20th International Conference.

[34] Li Q., Xiong R., Vidal-Calleja T.J.R., Systems A. (2017). A GMM based uncertainty model for point clouds registration. Robot. Auton. Syst..

[35] Fan J., Yang J., Ai D., Xia L., Zhao Y., Gao X., Wang Y. (2016). Convex hull indexed Gaussian mixture model (CH-GMM) for 3D point set registration. Pattern Recognit..

[36] Min Z., Wang J., Meng M.Q.-H. Robust generalized point cloud registration using hybrid mixture model. Proceedings of the 2018 IEEE International Conference on Robotics and Automation (ICRA).

[37] Shu Q., Fan Y., Wang C., He X., Yu C. (2021). Point Cloud Registration Algorithm Based on Laplace Mixture Model. IEEE Access.

[38] Tang Z., Liu M., Zhao F., Li S., Zong M. (2020). Toward a robust and fast real-time point cloud registration with factor analysis and Student’s-t mixture model. J. Real-Time Image Process..

[39] Forbes A. (2021). Approximate models of CMM behaviour and point cloud uncertainties. Meas. Sens..

[40] Peel D., McLachlan G.J. (2000). Robust mixture modelling using the t distribution. Stat. Comput..

[41] Basso F., Menegatti E., Pretto A. (2018). Robust intrinsic and extrinsic calibration of RGB-D cameras. IEEE Trans. Robot..

[42] Halmetschlager-Funek G., Suchi M., Kampel M., Vincze M. (2018). An empirical evaluation of ten depth cameras: Bias, precision, lateral noise, different lighting conditions and materials, and multiple sensor setups in indoor environments. IEEE Robot. Autom. Mag..

[43] Christian J.A., Cryan S. A survey of LIDAR technology and its use in spacecraft relative navigation. Proceedings of the AIAA Guidance, Navigation, and Control (GNC) Conference.

[44] Horaud R., Hansard M., Evangelidis G., Ménier C. (2016). An overview of depth cameras and range scanners based on time-of-flight technologies. Mach. Vis. Appl..

[45] Curless B., Levoy M. A volumetric method for building complex models from range images. Proceedings of the 23rd Annual Conference on Computer Graphics and Interactive Techniques.

